# A Facile Surface Modification Scheme for Medical-Grade Titanium and Polypropylene Using a Novel Mussel-Inspired Biomimetic Polymer with Cationic Quaternary Ammonium Functionalities for Antibacterial Application

**DOI:** 10.3390/polym16040503

**Published:** 2024-02-12

**Authors:** Chi-Hui Cheng, Xiang-Zhen Zeng, Wen-Yuan Chiu, Jui-Che Lin

**Affiliations:** 1 Department of Pediatrics, College of Medicine, Chang Gung University, Chang Gung Memorial Hospital, Taoyuan 33305, Taiwan; pedneph.cheng@msa.hinet.net; 2 Department of Chemical Engineering, National Cheng Kung University, Tainan 70101, Taiwan; q70w753753@gmail.com (X.-Z.Z.); chiu1999.vivian@gmail.com (W.-Y.C.); 3 Institute of Oral Medicine, College of Medicine, National Cheng Kung University, Tainan 70101, Taiwan; 4 School of Dentistry, College of Medicine, National Cheng Kung University, Tainan 70101, Taiwan

**Keywords:** quaternary ammonium, antibacterial, dopamine, mussel-inspired, surface modification

## Abstract

Medical device-associated infection remains a critical problem in the healthcare setting. Different clinical- or device-related methods have been attempted to reduce the infection rate. Among these approaches, creating a surface with bactericidal cationic functionality has been proposed. To do so, a sophisticated multi-step chemical procedure would be needed. Instead, a simple immersion approach was utilized in this investigation to render the titanium and polypropylene surface with the quaternary ammonium functionality by using a mussel-inspired novel lab-synthesized biomimetic catechol-terminated polymer, PQA-C8. The chemical oxidants, CuSO_4_/H_2_O_2_, as well as dopamine, were added into the novel PQA-C8 polymer immersion solution for one-step surface modification. Additionally, a two-step immersion scheme, in which the polypropylene substrate was first immersed in the dopamine solution and then in the PQA-C8 solution, was also attempted. Surface analysis results indicated the surface characteristics of the modified substrates were affected by the immersion solution formulation as well as the procedure utilized. The antibacterial assay has shown the titanium substrates modified by the one-step dopamine + PQA-C8 mixtures with the oxidants added and the polypropylene modified by the two-step scheme exhibited bacterial reduction percentages greater than 90% against both Gram-positive *S. aureus* and Gram-negative *E. coli* and these antibacterial substrates were non-cytotoxic.

## 1. Introduction

Despite the technological advances in clinical care, healthcare-associated infections (HAIs), also known as nosocomial infections, remain critical issues to patient health as well as healthcare costs. Among these HAIs, infections resulting from the inadvertent microbial adhesion onto the medical devices or materials used around hospital wards have received great attention from people in various disciplines, including material scientists, biomedical engineers, and clinical practitioners [1,2].

From the material science perspective, designing an antimicrobial surface to reduce microbial adhesion and/or kill the microbes before or after adherence has been the common approach to reduce material-related infections [3,4,5]. Among these approaches, killing the microbes before contact with the surface would rely on the bactericidal agents which were either released from the surface or added externally by various means. Nevertheless, time-dependent bactericidal efficacy and the emergence of biocide-resistant strains have hindered the wide use of bactericidal agents. Building a bactericidal surface, with cationic polymers such as pyridinium, phosphonium, and quaternary ammonium functionalities [6,7,8] to kill the microbes on contact has become an alternative approach due to its long endurance. Due to their excellent environmental stability, compounds with quaternary ammonium functionalities are the most popular selection [2,9,10]. Nevertheless, the safety of quaternary compounds as disinfectants has been of concern in the latest report [11]. To reduce the safety concerns about using the quaternary compounds in their free form, multiple processing steps or complicated/sophisticated chemical reactions would be needed to deposit a layer with quaternary ammonium functionalities covalently or tightly onto various surfaces that are commonly used in the medical industry, such as the titanium and polypropylene studied here.

Titanium and polypropylene were commonly used in different medical applications. Titanium or its alloys were seen in different orthopedic or dental applications, such as temporary and long-term external fixations, and craniofacial and dental implants [12,13], which generally required fairly tough physical properties. The medical applications of polypropylene, instead, were more widely noted in various clinical practices [14]. Polypropylene can be easily processed into different geometric shapes such as tubing, films, or even fibrous meshes used in hernia or pelvic organ prolapse [15,16]. Nevertheless, there were various endeavors to improve the biological interactions between the tissue and these two artificial synthetic materials. Improving the antimicrobial capability also attracted a vast amount of research interest including the use of functional deposits or surface functionalization of the titanium or polypropylene [17,18,19,20,21,22] with different surface modification techniques.

Inspired by mussels’ adhesion capability on various organic and inorganic surfaces in different aquatic environments, various biomimetic approaches based upon the mussels’ adhesion mechanisms have been attempted in different fields, such as membrane separation, dentin bonding, coatings, and various biomedical applications to name a few [5,23,24,25,26,27]. These investigations stemmed from the seminal findings of Messersmith’s team on the DOPA-inspired compound, dopamine’s adhesion onto different materials’ surfaces [28], and those of Caruso’s team on the natural polyphenol film formation [29] on various substrates. Depending upon the substrate and the preparation solution, the interactions between the substrate and the deposited dopamine, polydopamine, or polyphenol as well as the deposit itself could be attributed to various molecular interactions, such as π–π stacking, cation–π interactions, hydrogen bonding, covalent bonding, hydrophobic–hydrophobic interactions, etc. [30,31,32].

Despite such complicated interactions, the mussel-inspired pathway has been in research focus for modifying surface characteristics of different substrates. Several studies have taken the advantages of residual amine or hydroxyl groups in the pre-deposited polydopamine layer for subsequent grafting polymerization, such as using the surface-initiated atom transfer radical polymerization, to imbue the modified layer with different chemical characteristics [33,34,35]. On the other hand, using a monomer with a dopamine-like configuration, dopamine methacrylamide (DMA), to form copolymers with various functionalities before coating can also lead to distinctive thin films [36,37]. Additionally, using a dopamine-conjugated initiator, such as 2-bromoisobutyryl bromide (BIBB), to form copolymers with intended functionalities before coating could also lead to successful thin film deposition [38,39]. Other approaches, such as direct chemical grafting of polymers with a pre-deposited polydopamine layer [40,41], co-deposition using a solution mixture of polydopamine and a compound of interest [42,43,44,45], and sequential deposition using polydopamine as the first step followed by the compound of interest [44] have been attempted. In these approaches, the substrates used were mainly cover glasses or silicon wafers while medical application-relevant metals and polymers, such as the titanium and polypropylene used in this study, were rarely used.

In this study, a novel biomimetic catechol-terminated linear polymer with cationic quaternary ammonium functionalities was synthesized using SET-LRP (single-electron transfer living radical polymerization) [46,47,48,49] to avoid the free radical scavenger effect associated with the catechol end if the conventional free radical polymerization step is utilized [50]. Further, this SET-LRP approach could reduce the chance of polymer chain scission or incomplete deprotection in the deprotection step if the –OH functional groups are protected in the atom transfer radical polymerization (ATRP) step as shown in a study by He et al. [38].

Different facile surface modification/coating schemes were attempted in this study to change the surface characteristics and biological contact properties of medical-grade titanium and polypropylene, the two commonly used metallic and organic materials in medical devices, while bearing distinctive chemical natures on their surfaces, by a simple immersion approach using this novel mussel-inspired compound, as compared to previous laborious schemes reported for improving the antimicrobial characteristics of titanium [51,52] and polypropylene [53,54]. These schemes included the use of a simple one-step immersion method with different amounts of dopamine added into the novel synthesized cationic polymer solution. A two-step immersion method, in which the substrate was modified by the dopamine, then followed by the novel cationic polymer, was also attempted on the polypropylene substrate. Adding the oxidants CuSO_4_/H_2_O_2_ into the immersion solution was also explored since these oxidants have been indicated to increase the polydopamine deposition rate [55,56]. The surface characteristics, antimicrobial capability, and cytotoxicity of different modified specimens were investigated. And the optimal processing scheme for preparing the antibacterial titanium and polypropylene was identified.

## 2. Materials and Methods

### 2.1. Materials

The chemicals used in this study, namely dopamine hydrochloride (DA), methyl trifluoroacetate (MTFA), triethylamine (TEA), p-toluenesulfonic acid (PTSA, Ts-OH), 2,2-dimethoxypropane (DMP), calcium chloride, silica gel, potassium carbonate, tetrahydrofuran (THF), α-bromoisobutyryl bromide (BIBB), dichloromethane (DCM), trifluoroacetic acid (TFA), 2-(dimethylamino) ethyl methacrylate (DMAEMA), 1-bromooctane, acetonitrile (AeCN), copper(I) bromide, tris[2-(dimethylamino)ethyl] amine (Me_6_TREN), tris(hydroxymethyl)aminomethane (Trizma base), hydrogen peroxide, and copper(II) sulfate pentahydrate were purchased from different vendors, including Sigma-Aldrich (St Louis, MO, USA), Alfa Aesar (Ward Hill, MA, USA), J.T. Baker (Phillipsburg, NJ, USA), SHOWA (Chuo-ku, Japan), Fluka (Milwaukee, WI, USA), MACRON (Phillipsburg, NJ, USA), and DUKSAN (Gyeonggi, Republic of Korea), at the highest purity available.

### 2.2. Synthesis of PQA-C8

**Scheme 1:**
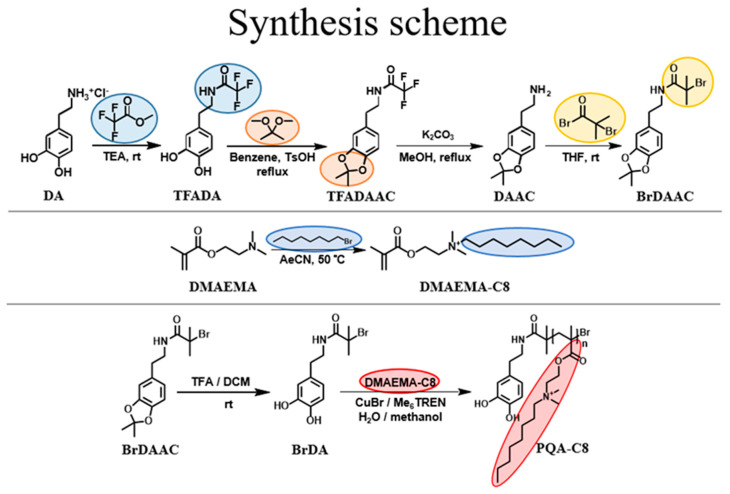
The synthesis scheme for the polymeric quaternary ammonium salt with a catechol terminal end, PQA-C8.

The overall synthesis scheme of the polymeric quaternary ammonium salt with a catechol terminal end, PQA-C8, is shown in Scheme 1.

#### 2.2.1. Synthesis of TFADA

A total of 20 g dopamine-HCl (105 mmol) was dissolved in 350 mL methanol and added into a round-bottom flask under argon protection. Then, 23 mL methyl trifluoroacetate (MTFA, 229 mmol) and 64 mL triethylamine (TEA, 459 mmol) were added into the flask. The reaction was carried out at room temperature for 24 h. After removal of the solvent, 1 N HCl(aq) was added to adjust the pH value to 3–4; the mixture was then extracted with ethyl acetate several times, washed with brine, dried over Na_2_SO_4_, the solvent was removed, and the compound was finally dried in vacuum. The brown, solid TFADA was obtained.

#### 2.2.2. Synthesis of TFADAAC

To protect the catechol group, the following step was carried out. A total of 7 g TFADA (28 mmol) was dissolved in 400 mL benzene and added into a three-neck round-bottom flask, and 15 mL 2,2-dimethoxypropane (DMP, 122 mmol) was added into the flask. A Soxhlet extractor was used to remove the byproducts. The thimble in the Soxhlet was filled with 30 g anhydrous CaCl_2_ to absorb water and methanol. After the first reflux, 0.2167 g *p*-toluenesulfonic acid (TsOH, PTSA, 1.38 mmol) was added into the flask. The reaction was carried out overnight and could be monitored by the FeCl_3_ test on a TLC plate. The solvent was then removed and passed through silica gel chromatography with dichloromethane. The final product was recrystallized in hexane and dried in a vacuum. The white, crystalline TFADAAC was obtained.

#### 2.2.3. Synthesis of DAAC

A total of 6 g TFADAAC (20.7 mmol) was dissolved in 180 mL methanol and added into a round-bottom flask. A total of 8.58 g of K_2_CO_3_ (62.1 mmol) and 5 mL of deionized (DI) water were added to the flask. The solution was heated up to reflux temperature and the reaction was monitored with a TLC plate. After filtration of the mixture and removal of the solvent, the product was dissolved in DI water and extracted with chloroform several times, washed with DI water, dried over Na_2_SO_4_, the solvent was removed, and the compound was dried in a vacuum overnight.

#### 2.2.4. Synthesis of BrDAAC

A total of 2 g DAAC (10.4 mmol) was dissolved in 20 mL tetrahydrofuran (THF) and added into a two-neck round-bottom flask. A total of 2.02 mL triethylamine (TEA, 14.5 mmol) was added into the flask and purged with argon for 20 min. The mixture was cooled in an ice bath, and then 1.343 mL α-bromoisobutyryl bromide (BIBB, 10.9 mmol) was added. The reaction was carried out at room temperature overnight. The crude product was then mixed with ethyl acetate for precipitation. The precipitate was then filtered and washed with 1 N NaHCO_3(aq)_, DI water, and brine, subsequently, and then dried over Na_2_SO_4_. Further purification by silica gel column chromatography with DCM as an eluent was undertaken. The solvent was removed and dried under vacuum, and the light brown, solid BrDAAC was obtained.

#### 2.2.5. Synthesis of BrDA

A total of 2 g BrDAAC (5.85 mmol) was first dissolved in 75 mL dichloromethane (DCM) and then added into a round-bottom flask and cooled in an ice bath for 15 min. Then, 25 mL trifluoroacetic acid (TFA) was added into the flask. The reaction was carried at room temperature for 6 h. After removing the solvent by a rotary evaporator, the dark brown, viscous liquid BrDA was obtained. 

#### 2.2.6. Synthesis of DMAEMA-C8

The quaternization reactions were carried out according to Wan et al. [57]. A total of 10 mL of 2-(dimethylamino) ethyl methacrylate (DMAEMA, 59.3 mmol) was in a 250 mL round-bottom flask equipped with a magnetic stirrer, wherein 11.277 mL 1-bromooctcane (65.6 mmol) was then added into the solution. Subsequently, approximately 30 mL of acetonitrile (AeCN) was added into the flask as solvent. The mixture was stirred at 60 °C for four days. A white powder was obtained after removing the solvent, then washed with anhydrous ether several times, and dried under a vacuum.

#### 2.2.7. Synthesis of PQA-C8

The SET-LRP synthesis procedure was modified from Zhang et al. and Guo et al. [46,47]. A total of 26 μL tris[2-(dimethylamino)ethyl]amine (Me_6_TREN, 0.1 mmol) and 500 μL H_2_O were added into a Schlenk tube (or flask) and were bubbled with argon for 5 min. A total of 14 mg CuBr was then added into the tube under a slight positive pressure of argon and this was kept bubbling for a further 10 min. The suspension turned purple and was set to keep stirring under an ice/water bath for 15 min. Meanwhile, 1 mL H_2_O, 4 mL methanol, 75 mg BrDA (0.25 mmol), and 1312 mg DMAEMA-C8 (3.75 mmol) were added into another tube and bubbled with argon for 15 min. After bubbling, the initiator/monomer aqueous solution was transferred to the other tube with Cu(0)/CuBr_2_/Me_6_TREN via a cannula. The polymerization was carried out for an hour, then dialyzed against water for 2 days, and freeze-dried to remove the water.

### 2.3. Surface Modification of Different Substrates

The titanium plates (AcrUshin Co., Tokyo, Japan) and polypropylene (PP) sheets (Tzefeng Plastics, Kaohsiung, Taiwan) were cut into small pieces and then cleaned with neutral detergent, deionized water, ethanol, and acetone under sonication several times. The titanium plates were stored in methanol while the PP sheets were dried and stored under vacuum. The titanium plates were further cleaned in piranha solution for 1 h and rinsed with deionized water before coating.

These substrates were immersed in the 10 mg/mL PQA-C8 in ethanol–tris buffer solution (1:1 volume ratio, 10 mM pH = 8.5) at different ratios of dopamine for 24 h, or 2 h if the oxidants were added in the reaction, at room temperature (Table 1). The concentration of oxidants, if used, was 1.25 mg/mL and 0.67 mg/mL for CuSO_4_ and H_2_O_2_, respectively. Subsequently, the substrates were ultrasonically washed with deionized water and dried with argon, then stored in vacuum.

In contrast to the one-step immersion scheme above, a two-step method was used to modify the PP substrates, in which the PP substrates were immersed into the dopamine solution first, followed by the PQA-C8 solution (Table 2). The final samples were ultrasonically cleaned as those coated by the one-step method were.

### 2.4. Characterization

The chemical configurations of PQA-C8 and the intermediates in the synthesis scheme were confirmed by nuclear magnetic resonance (NMR) (Bruker AVNEO 500NMR or AVIIIHD700, Fällanden, Switzerland). Various surface characteristics, including surface hydrophobicity, surface morphology, and chemical bonding state and element composition of the modified surfaces were analyzed by water contact angle (WCA) measurements (Model 100SB, Sindatek Inc., Taipei City, Taiwan), scanning electron microscopy (SEM) (SU8010, Hitachi, Tokyo, Japan), and X-ray photoelectron spectroscopy (XPS) (PHI Quantera II, ULAVAC-PHI, Chigasaki, Japan), respectively.

### 2.5. Antibacterial Test

Since the density of modified PP was less than that of water, instead of using the flat flask, the antibacterial test was performed by placing the nascent or modified substrates into the glass tubes tightly while not touching the round bottom of the tubes. The bacteria (*S. aureus*, ATCC 21351, and *E. coli,* ATCC 23501) were first cultivated in Luria–Bertani medium for 24 h at 37 °C. After serial dilutions, 1 mL of bacteria suspension (2 × 10^6^ CFU/mL) was added into each tube that contained the substrates and incubated for 6 h at 37 °C under 150 rpm of shaking. The substrates were fully immersed in bacteria solution throughout this incubation duration. Previous studies have indicated that UV irradiation could enhance the bactericidal capability as well as lead to a significant reduction in bacterial attachment and subsequent biofilm formation on titanium dioxide surfaces, an oxidized layer commonly found on the titanium substrate [58,59]. Henceforth, the antibacterial test was performed *“in the dark”* to avoid the interference of light that may change the interactions of the microbes and the unmodified/modified Ti and PP substrates. After that, the samples were ultrasonicated for 5 min to detach the adhered bacteria on the substrates. Last, the bacteria suspension in the tube was removed, diluted, and spread onto agar plates to count the colonies to measure the viability of the bacteria.

### 2.6. Cytotoxicity Assay

L929 mouse fibroblast cells (NCTC clone 929, BCRC-RM60091) were used in the cytotoxicity test. According to the standard ISO 10993-5 and ISO 10993-12 protocols, the cytotoxicity test was conducted by the extraction method. The L929 cell suspension was cultured in Minimum Essential Medium (MEM) containing 10% horse serum (HS), 1% penicillin–streptomycin (P/S), 1% HEPES solution, 1% MEM non-essential amino acid solution (100x), 1% sodium pyruvate solution, and 1% GlutaMAX^TM^-1 (100x) at 37 °C and 5% CO_2_, and the medium was replaced every three days. The testing substrate was first sterilized by soaking it in 75% ethanol. The testing substrates (n = 5) were then immersed in the culture medium and incubated for 24 h. The L929 cells (density = 10^4^ cells/well) were seeded in a 96-well plate and cultured for 24 h at 37 °C and 5% CO_2_ atmosphere. The medium was replaced with the eluent from the substrate after 24-h medium incubation and then incubated at 37 °C and 5% CO_2_ atmosphere for 24 h. The cell viability was determined by the MTT assay. An Enzyme-Linked Immunosorbent Assay (ELISA) was used to detect the absorption at 570 nm. The reference wavelength was set at 650 nm. A polyethylene plastic wrap and a latex glove were used as the negative and positive controls for the cytotoxicity testing.

### 2.7. Copper Ion Release Test

To confirm the relationship between the possible released copper ions and cytotoxicity assay results, an inductively coupled plasma mass spectrometer (ICP-MS) (THERMO-ELEMENT XR, USA) was used to measure the copper ion concentration released if the samples were prepared with the CuSO_4_/H_2_O_2_ oxidants added. Each sample was immersed in 5 mL PBS at room temperature under 150 rpm for 3 days. After that, the samples were taken out, then the copper ion concentration in the PBS was measured by ICP-MS.

### 2.8. Statistical Analysis

All the quantitative analyses were repeated at least three times except the XPS analyses, and the results are expressed as mean ± standard deviation. Quantitative comparisons using Student’s *t*-testing with a *p*-value of less than 0.05 were considered statistically significant.

## 3. Results and Discussion

### 3.1. PQA-C8 Synthesis

The chemical configuration of the lab-prepared PQA-C8, the final polymeric quaternary ammonium compound with catechol terminal ends, and the intermediates including the TFADA, TFADAAC, DAAC, BrDAAC, BrDA, and DMAEMA-C8 shown in Scheme 1, were analyzed by ^1^H-NMR spectroscopy (Figures S1–S7). The NMR spectra indicated all these compounds were well prepared at >90% purity.

### 3.2. Surface Characterization

#### 3.2.1. Surface Morphology

##### Surface Morphology of the Modified Titanium Substrates

The surface morphologies of the bare titanium and the titanium substrates modified with different methods are shown in Figure S8. The bare titanium presented few pits and a coral-like structure, likely resulting from the piranha cleaning/oxidation/etching effect after immersion for 1 h. After immersion into the PQA-C8 solution with or without the dopamine, while no oxidants were added, various submicron or micron sizes of aggregates/deposits were noted on the titanium substrates. Further, with more dopamine added, a less particulate-like structure was noted on the modified titanium substrate (i.e., 10:1-Ti vs. 10:0.5-Ti vs. 10:0-Ti). After adding the oxidants, CuSO_4_/H_2_O_2_, into the PQA-C8 + dopamine solution, the titanium substrate surface presented larger aggregates as compared to their counterparts (e.g., 10:0.5-Ti (CuSO_4_/H_2_O_2_) vs. 10:0.5-Ti) even at a shorter immersion duration (2 h vs. 24 h). This may be attributed to the enhanced formation of PQA-C8 + dopamine aggregates or polymers in the solution before being attached/deposited onto the titanium substrate.

##### Surface Morphology of the Modified Polypropylene Substrates

Various sub-micron pits/holes were noted on the bare PP surface (Figure S9), likely resulting from the processing effects on the PP sheet formation. After immersion into the PQA-C8 + dopamine solution in different weight ratios, without or with the oxidants CuSO_4_/H_2_O_2_, surface deposits were noted on these one-step process-modified PP substrates; they exhibited different surface roughness or aggregates. For the PP substrate modified by the 2-step process, DA-PP, larger aggregates were noted as compared to the bare PP. Further immersion of the DA-PP substrate into the PQA-C8 solution led to even larger aggregates on the PQA-DA-PP surface. This suggested that the surface deposits were successfully formed on the PP substrates using the 2-step process scheme.

#### 3.2.2. Surface Hydrophilicity

##### Surface Hydrophilicity of the Modified Titanium Substrates

**Figure 1:**
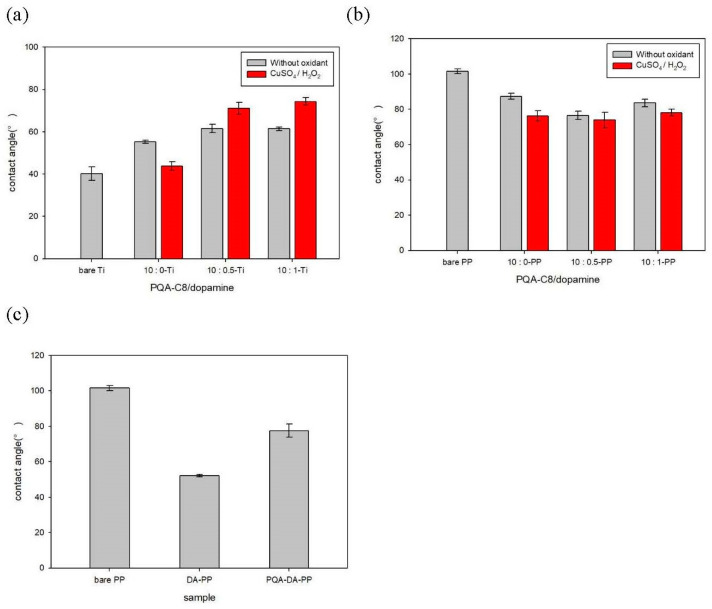
Static contact angle value of the one-step-modified (**a**) titanium, (**b**) PP substrates, and two-step-modified (**c**) PP substrates.

For the titanium substrate modified by the one-step deposition process without using the dopamine and oxidants (10:0-Ti) (Figure 1a), the contact angle was increased (*p* < 0.05) after 24-h immersion in PQA-C8, likely resulting from the hydrophobic alkyl chains associated with PQA-C8. In contrast, the contact angle of the PQA-C8-modified titanium, prepared with the oxidants while without the dopamine addition (10:0-Ti CuSO_4_/H_2_O_2_), remained similar to the untreated bare titanium control (*p* > 0.05). This may be attributed to the added oxidants hindering the transformation of surface Ti-OH formed after piranha solution cleaning to the Ti-O-Ti that can facilitate the surface bonding of PQA-C8 through the quinone end, the terminal end formed after oxidation of catechol [60]. Reduced transformation of the catechol end to the quinone structure due to the bulky cationic polymer end, as compared to the dopamine, could likely contribute to this finding. Nevertheless, incomplete coverage on the titanium substrate (see Section 3.2.3 XPS analysis) due to the shorter immersion time (2 h vs. 24 h) may lead to this bare-Ti-similar contact angle finding as well.

Adding dopamine to the PQA-C8 immersion solution resulted in a further increase in surface hydrophobicity (10:0.5-Ti and 10:1-Ti), either without or with the oxidants. Nevertheless, the contact angle did not vary significantly (*p* > 0.05) with the amount of dopamine added (Figure 1a). Further, the substrates prepared with the oxidants added presented a higher surface hydrophobicity than their counterparts, e.g., 10:0.5-Ti (CuSO_4_/H_2_O_2_) vs. 10:0.5-Ti, even with a shorter immersion duration. This is likely attributed to the enhancement in PQA-C8 deposition when dopamine was added. In addition, adding the CuSO_4_/H_2_O_2_ oxidants could transfer the catechol configuration in dopamine to the quinone structure, leading to a further increase in surface hydrophobicity [55].

##### Surface Hydrophilicity of the Modified Polypropylene Substrates

In contrast to the titanium substrate, the one-step PQA-C8-modified PP without CuSO_4_/H_2_O_2_ oxidants showed lower contact angle values than the bare hydrophobic PP substrates (*p* < 0.05), likely resulting from the incorporation of quaternary ammonium functionalities (Figure 1b). This implicated that PQA-C8 was deposited on the PP substrate. Adding dopamine into the PQA-C8 solution at different ratios without CuSO_4_/H_2_O_2_ oxidants led to lower contact angle values (*p* < 0.05). This suggested that dopamine could enhance the PQA-C8 deposition, as noted on the titanium substrate. However, the contact angle could increase if a higher amount of dopamine is added (10:1 PP vs. 10:0.5-PP, *p* < 0.05). In contrast, adding dopamine into the PQA-C8 solution with CuSO_4_/H_2_O_2_ oxidants did not significantly change the contact angle (*p* > 0.05). The degree of contact angle variation is apparently influenced by the substrate utilized. When the CuSO_4_/H_2_O_2_ oxidants were added to the PQA-C8 + dopamine solution, the surface hydrophobicity variation on the modified PPs was quite distinct from that on the titanium. This further substantiated the fact that the deposition process would be greatly affected by the substrate’s chemical and/or physical characteristics.

In contrast to the one-step immersion scheme for modifying the PP substrates, a two-step modification scheme was explored based on the hypothesis that dopamine can enhance PQA-C8 deposition, noted from the contact angle findings in the one-step process. Previous studies have suggested that a deposited polydopamine layer can serve as the anchor for subsequent deposition on various substrates [61,62,63,64]. The two-step modification scheme utilized here was immersing the substrate into the dopamine solution first and then immersing in the PQA-C8 solution. This two-step modification scheme was not attempted on the titanium substrate since the surface titanium oxide layer on the Ti substrate (see Section 3.2.3 XPS analysis) can be bound with the catechol functionalized polymers by chemisorption [60]. In contrast, the PP surface presented only a small number of oxygen-containing functionalities, likely the hydroxyl groups (see Section 3.2.3 XPS analysis, Table 4). Henceforth, to overcome this problem, the polymeric substrates may need to be modified before the PQA-C8 deposition. In this regard, the two-step modification scheme was explored.

Instead of using an acidic chemical etchant or plasma to add the specific chemical functionalities needed, the PP substrates were immersed into the dopamine solution followed by the PQA-C8 solution. This gentle immersion approach could reduce the chance of deterioration of physical properties in polymeric substrates that could occur if plasma or chemical etchants were used.

For the PP substrate modified by the two-step modification scheme, the surface contact angle value decreased significantly (*p* < 0.05) after first immersion into the dopamine solution (i.e., DA-PP in Figure 1c). This could be attributed to the polydopamine deposited on the hydrophobic PP substrates. The surface contact angle increased (*p* < 0.05) with the additional PQA-C8 immersion (i.e., PQA-DA-PP), likely due to the long C8 alkyl chains on the PQA-C8. Subsequent surface XPS analysis (see Section 3.2.3 XPS analysis) would further delineate the differences in surface chemical characteristics of these modified PPs.

#### 3.2.3. XPS Analysis

The surface atomic percentages of different modified titanium and PP substrates are shown in Table 3 and Table 4, respectively. The quaternary N^+%^ was determined by N1s atomic percentage times; the C-N^+^ area percentage value was derived from the N1s curve fitting. The N1s peak was deconvoluted to the C-N (399.8 eV) and C-N^+^ (402.4 eV) for all samples except the bare Ti, in which it was deconvoluted to the Ti-N (397 eV) and C-N (399.8 eV) [65,66] (Figures S10 and S11).

##### Surface Chemical Characteristics of the Modified Titanium Substrates

For the bare Ti substrate, C1s, N1s, and O1s were noted and it likely resulted from the adsorbed adventitious hydrocarbons during the Ti plate preparation process (Table 3). The Ti2p atomic percentage was greatly reduced after modification with PQA-C8 + dopamine solution without or with the addition of CuSO_4_/H_2_O_2_ oxidants as compared to the ones modified with the PQA-C8 only. Additionally, an increase in C1s and N1s atomic percentages and a decrease in O1s atomic percentage were noted in such a comparison. This indicated that a layer of mixed dopamine + PQA-C8 deposit was formed on the Ti surface. Further, the N1s and quaternary N^+^ percentage increased with more dopamine added into the immersion mixture without or with the CuSO_4_/H_2_O_2_ oxidants. This suggested that the addition of dopamine into the deposition solution could assist the deposition of PQA-C8 onto the Ti substrate.

Nevertheless, the decreases in the Ti2p and O1s atomic percentages and increases in N1s and quaternary N^+^ percentages were far lower when the CuSO_4_/H_2_O_2_ oxidants were added into the deposition solution as compared to their counterparts. This can likely be attributed to incomplete deposit coverage due to the shorter deposition duration and/or the chemical effects exerted by the oxidants on the Ti substrate and PQA-C8 as described in Section 3.2.2, surface hydrophilicity. Further, a Cu2p signal was noted on these oxidants-added modified Ti samples despite ultrasonic cleaning after deposition treatment, likely resulting from the chelation of copper with dopamine or the catechol chemical configuration [45].

To further delineate the surface chemical characteristics of the deposit, the C1s spectra of different titanium substrates were deconvoluted [67,68]. The Ti-C noted on the bare Ti substrate, likely resulting from the Ti preparation process, disappeared completely after immersion into the deposition solution (Figure S12 and Table S1). Further, all PQA-C8 + doapmine-modified Ti substrates, with or without CuSO_4_/H_2_O_2_ oxidants, exhibited all carbon bonds noted in the PQA-C8 and dopamine chemical configuration and the relative percentages of these bonds did not vary much (Table S1).

##### Surface Chemical Characteristics of the Modified Polypropylene Substrates

For the PP substrates modified by the one-step deposition process in the non-oxidant-added system, in opposition to the findings noted for the titanium substrates (Table 3), the N1s and quaternary N^+^ percentage values on the modified PP substrates were quite low for samples modified with PQA-C8 solution only (i.e., 10:0-PP, Table 4). Adding dopamine into the deposition solution in the non-oxidant-added system enhanced the PQA-C8 deposition onto the PP substrate. This finding further highlights that the surface chemical and/or physical characteristics of the bare substrate could play important roles, yet to be determined, in the solution deposition process.

Adding the CuSO_4_/H_2_O_2_ oxidants into the PQA-C8 + dopamine deposition solution could increase the surface N1s and N^+^ atomic percentage values. In the meantime, unlike the titanium substrate, the Cu2p atomic percentage was very low on these modified PP substrates. This may implicate less dopamine/catechol chemical structure bound to the PP substrate as compared to the titanium one.

The surface chemical composition of the PP modified by a two-step process was also analyzed (PQA-DA-PP in Table 4). The PQA-DP-PP presented similar surface chemical atomic percentages as those modified by the one-step process with dopamine and oxidants (CuSO_4_/H_2_O_2_) added, but without the Cu2p signals.

The C1s spectra of the PP substrates modified by the one-step and the two-step process were curve-fitted as well (Figure S13 and Table S2) [67,68]. The C-C/C-H area percentage decreased after the PP was modified and the least value was noted on the PQA-DA-PP substrate, implicating the two-step process was likely the most effective process to deposit the PQA-C8 onto the PP surface. Additionally, adding the dopamine into the PQA-C8 deposition solution, with or without adding the CuSO_4_/H_2_O_2_ oxidants, can lead to an increase in C=O area percentage and a decrease in C-C/C-H area percentage as compared to the ones of samples modified by the pure PQA-C8 solution. This suggested that dopamine can assist the PQA-C8 deposition onto the PP substrate.

### 3.3. Antibacterial Assay

The antibacterial activity, as determined by the bacterial reduction percentage, of differently modified samples was first examined against Gram-positive *S. aureus* (Table 5). Although contact angle and XPS analyses implicated that adding dopamine can assist the PQA-C8 deposition on titanium substrates, the one modified by the PQA-C8 only (i.e., 10:0-Ti) presented the highest bacterial reduction percentage in the non-oxidant-added system (10:0-Ti vs. 10:0.5-Ti vs. 10:1-Ti; *p* < 0.05). This may be due to the surface orientation of the bactericidal cationic terminal functionalities, as well as the neighboring chemical environment around the quaternary functionalities yet to be determined. In contrast, adding the CuSO_4_/H_2_O_2_ oxidants to the immersion solution greatly improved the antibacterial capability of the modified titanium substrates if the dopamine was added into the PQA-C8 solution (i.e., no statistical difference was noted between 10:0-Ti and 10:0-Ti (CuSO_4_/H_2_O_2_); *p* > 0.05). This may be partially attributed to the surface Cu ions noted by the XPS analyses (Table 3). Nevertheless, changes in the surface chemical configuration and composition of these CuSO_4_/H_2_O_2_ oxidant-modified titanium substrates should not be overlooked and extra experimental works are warranted to elucidate these findings.

For the one-step-modified PP, only the PP substrates modified using the CuSO_4_/H_2_O_2_ oxidants showed a fair antibacterial capability against *S. aureus*. Although dopamine can assist the PQA-C8 deposition onto the PP substrates (Table 4), the antibacterial capability of the modified PP substrate was not much improved without adding CuSO_4_/H_2_O_2_ oxidants, implicating these surfaces may not have the right surface configuration needed for antibacterial applications. Nevertheless, the detailed causes remain to be explored.

The PP modified by a two-step process, instead exhibited a good antibacterial capability against *S. aureus*. This implicated that the dopamine deposited in the first step did not only enhance the following PQA-C8 deposition (Table 4) but also enhanced the right antibacterial surface configuration needed against *S. aureus.*

The modified samples with greater than 90% bacterial reduction percentage against *S. aureus* were further tested against Gram-negative *E. coli* (Table 5). The titanium substrates modified with the solution added with CuSO_4_/H_2_O_2_ oxidants and the PP modified by the two-step process also had a good antibacterial capability against *E. coli* while the others did not. These findings were in accord with a previous study in which Gram-negative microbes were less likely to be killed by a surface with quaternary ammonium functionalities than Gram-positive ones [69]. Nevertheless, other surface characteristics, such as the density of cationic quaternary ammonium functional groups, and others that needed to be further explored, could also lead to different antimicrobial capabilities against microbes with different Gram stains noted here.

### 3.4. Cytotoxicity Assay

**Figure 2:**
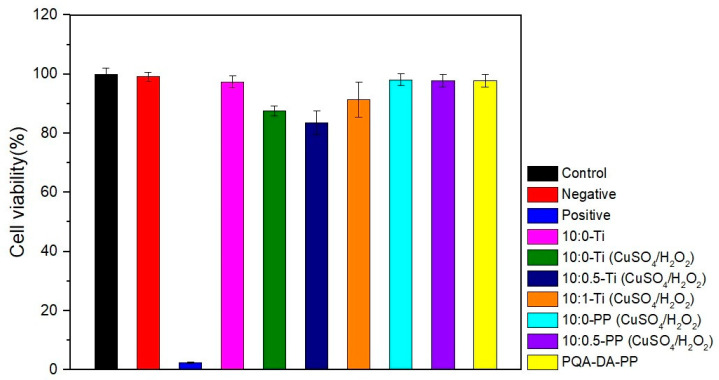
Cytotoxicity assay of different samples (n = 3).

Cytotoxicity assessment following the ISO 10993-5 and ISO 10993-12 protocols was performed on the modified samples with greater than 90% bacterial reduction percentage against *S. aureus*. Samples that showed greater than 70% cell viability were considered non-cytotoxic [70,71]. Figure 2 demonstrates that all samples were non-cytotoxic, with more than 80% cell viability.

### 3.5. Copper Ion Release Test

Since copper ions were reported to be cytotoxic, it is imperative to determine the concentrations of copper ions released when the samples were modified with the use of CuSO_4_/H_2_O_2_ as the oxidative reagents. After immersion in PBS solution for 3 h, the released copper ion concentrations for the samples tested for cytotoxicity (Figure 2) were determined. It was noted that the release of copper ions was quite low in any case (Table S3). This finding was in accord with the low cytotoxicity values noted in Figure 2. This finding further suggested that the higher antibacterial activity noted on these CuSO_4_/H_2_O_2_ oxidant-modified samples (Table 5) could be partially attributed to surface, not released, copper ions.

### 3.6. Discussion

Improving the antibacterial capability of artificial synthetic biomaterials remains a challenging task, especially from the perspective of ease of production process as well as the cost associated with the process. This study has indicated the antibacterial capability of metallic titanium and organic polypropylene can be improved by surface modification with a novel mussel-inspired catechol-terminated cationic polymer with quaternary ammonium functionalities by simple immersion methods using different immersion solution formulations and processing methodologies. This finding may guide the future direction in selecting the immersion process conditions/parameters for improving the surface antimicrobial characteristics of other existing metallic and organic FDA-approved biomaterials with this novel cationic polymer.

The antimicrobial capabilities of the titanium and polypropylene modified by the optimum conditions were comparable to those reported [18,72,73]. Nevertheless, this investigation is only the beginning of screening the processing conditions for the optimum antibacterial characteristics against the two common pathogenic microbes *S. aureus* and *E. coli*. Further antimicrobial evaluation should be tailored based on the final clinical applications. For example, a clinically relevant flow field should be utilized if the polypropylene is tubular. Other microbes, such as *Porphyromonas gingivalis* and *Prevotella intermedius/nigrescens* needed to be examined if the titanium is used for dental implants since these microbes were commonly noted in peri-implantitis sites [74].

The novel catechol-terminated cationic polymer synthesized here can be considered as one of the quaternary ammonium compounds (QACs). Despite their common use as disinfectants, the safety of QACs has been of concern in the latest report [11]. This study utilized processing schemes to ensure this cationic polymer can be covalently or tightly bound to the titanium and polypropylene substrates. Henceforth, the cytotoxicity assay using the eluent from these modified substrates based upon the ISO 10993-5 and ISO 10993-12 standards has shown non-cytotoxicity against L929 mouse fibroblast cells. Nonetheless, once the final clinical application is determined, cytotoxicity against the human cells which direct contact to the modified materials, such as uroepithelium cells in urinary tube applications or osteoblasts and human fibroblast cells in dental/orthopedic uses, should be examined using both eluent and direct surface-contact methods to ensure these cationic polymer-modified materials are safe. Advanced, clinically relevant animal models are still warranted to confirm these modified materials can be of use in the final clinical setting.

The process scheme to prepare the optimum antimicrobial surface was quite different between the metallic titanium and organic polypropylene substrates. For the titanium substrate, a simple one-step immersion scheme can greatly improve the antimicrobial capability using the solution containing the novel cationic polymer, different amounts of dopamine, and oxidants. In contrast, for the polypropylene substrate, this one-step immersion scheme did not improve the antimicrobial capability against both *S. aureus* and *E. coli*. Rather, a two-step scheme, in which the dopamine was first deposited, followed by the deposition of the novel catechol-containing cationic polymer, can greatly increase the antibacterial properties of polypropylene. This implicated the importance of the substrate’s surface properties in governing the surface deposition process, either through chemical reactions or strong physical bonding, such as hydrogen bonds or electrostatic interactions. For a poly-olefinic substrate, such as the polypropylene studied here, the virgin surface may not have the desired characteristics or surface functionalities needed for proper deposition of this catechol-containing cationic polymer. A pre-deposited polydopamine layer could enhance the deposition and orientation of cationic functionalities that are needed for greater antimicrobial capability. Such a polydopamine-assisted grafting/deposition process has been proposed in various studies [61,62,63,64,75,76] and, henceforth, a similar two-step immersion scheme would be the likely choice for improving the antimicrobial properties of substrates not having the reactive functionalities towards this novel catechol-terminated cationic polymer, for example polyethylene (PE), polyvinyl chloride (PVC), or even elastic polystyrene–butadiene–styrene rubber (SBS).

## 4. Conclusions

A novel polymer with cationic quaternary ammonium functionalities and a biomimetic catechol terminal end, PQA-C8, was successfully synthesized. This polymer was employed for mussel-inspired surface modification of medical-grade titanium and polypropylene using a one-step or a two-step immersion scheme. Different amounts of dopamine were added into the PQA-C8 ethanol–tris buffer solution (pH = 8.5), with or without the use of CuSO_4_ and H_2_O_2_ oxidants, as the immersion solution for the one-step modification scheme. Contact angle and XPS analyses indicated that, using the one-step modification process, dopamine would assist the PQA-C8 deposition onto the titanium and polypropylene with or without the CuSO_4_ and H_2_O_2_ oxidants. Using the two-step modification scheme, in which the dopamine was deposited onto the plastic substrate before the PQA-C8, can easily change the surface properties of polypropylene.

The antibacterial assay revealed that only a few modified substrates, namely the one-step dopamine + PQA-C8-modified titanium substrates with the oxidants added and the two-step modified polypropylene, exhibited bacterial reduction percentages greater than 90% against both Gram-positive *S. aureus* and Gram-negative *E. coli*. This highlighted the importance of surface configuration/surface composition of the deposited PQA-C8-containing layer on different modified substrates in governing the material’s antibacterial activity. Further experiments are warranted to further elucidate these interrelationships as well as large-scale/industrial-scale production of antibacterial surfaces with such simple immersion schemes.

## Figures and Tables

**Table 1:** Sample nomenclature for those coated via the one-step method. X: Ti or PP.

Sample (X = Ti, PP)	Conc. of PQA-C8	Conc. of Dopamine	Oxidants and Coating Duration
Bare X	Cleaning only		
10:0-X	10 mg/mL	-	Nil The coating duration was 24 h
10:0.5-X	10 mg/mL	0.5 mg/mL	
10:1-X	10 mg/mL	1 mg/mL	
10:0-X (CuSO_4_/H_2_O_2_)	10 mg/mL	-	CuSO_4_/H_2_O_2_ The coating duration was reduced to 2 h
10:0.5-X (CuSO_4_/H_2_O_2_)	10 mg/mL	0.5 mg/mL	
10:1-X (CuSO_4_/H_2_O_2_)	10 mg/mL	1 mg/mL	

**Table 2:** Sample nomenclature for those coated via the 2-step method.

Sample	Treatment
Bare PP	Cleaning only
DA-PP	Cleaning followed by immersion in 2 mg/mL of dopamine solution (10 mM tris buffer) for 4 h
PQA-DA-PP	The DA-PP was further immersed in 10 mg/mL of PQA-C8 solution (ethanol–tris buffer; 1:1 volume ratio, 10 mM pH = 8.5) for 24 h

**Table 3:** Surface atomic percentage of titanium substrate modified by different methods.

Sample	Ti2p	C1s	N1s	O1s	Cu2p	N^+^
Bare Ti	18.8%	30.7%	2.9%	47.6%	-	0%
10:0-Ti	8.1%	51.3%	3.7%	36.9%	-	2.34%
10:0.5-Ti	1.6%	69.5%	5.8%	23.2%	-	2.87%
10:1-Ti	0.7%	71.7%	6.1%	21.5%	-	3.06%
10:0-Ti (CuSO_4_/H_2_O_2_)	11.9%	41.3%	2.8%	40.4%	3.6%	1.77%
10:0.5-Ti (CuSO_4_/H_2_O_2_)	8.9%	50.6%	3.3%	35.0%	2.2%	1.65%
10:1-Ti (CuSO_4_/H_2_O_2_)	5.9%	58.0%	4.2%	29.1%	2.7%	2.81%

**Table 4:** Surface atomic percentage of polypropylene substrate modified by different methods.

Sample	C1s	N1s	O1s	Cu2p	N^+^
Bare PP	93.7%	0.7%	5.6%	-	0%
10:0-PP	82.1%	0.8%	17.1%	-	0.56%
10:0.5-PP	80.0%	3.5%	16.6%	-	2.47%
10:1-PP	79.7%	4.1%	16.2%	-	2.24%
10:0-PP (CuSO_4_/H_2_O_2_)	88.6%	1.7%	9.3%	0.5%	1.31%
10:0.5-PP (CuSO_4_/H_2_O_2_)	76.7%	3.9%	19.0%	0.4%	2.93%
10:1-PP (CuSO_4_/H_2_O_2_)	79.1%	3.3%	17.5%	0.2%	2.65%
PQA-DA-PP	76.2%	4.1%	19.6%	-	2.67%

**Table 5:** The bacterial reduction percentage of different samples against *S. aureus* and *E. coli* (n = 3).

Sample	Bacterial Reduction (%)	
	*S. aureus* (ATCC 21351)	*E. coli* (ATCC 23501)
10:0-Ti	97.7 ± 2.4	72.28 ± 13.8
10:0.5-Ti	64.8 ± 12.5	-----
10:1-Ti	46.0 ± 4.7	-----
10:0-Ti (CuSO_4_/H_2_O_2_)	98.1 ± 0.5	98.49 ± 0.6
10:0.5-Ti (CuSO_4_/H_2_O_2_)	98.4 ± 0.6	98.23 ± 0.8
10:1-Ti (CuSO_4_/H_2_O_2_)	98.0 ± 1.5	99.25 ± 0.8
10:0-PP	49.8 ± 4.5	-----
10:0.5-PP	68.5 ± 5.8	-----
10:1-PP	54.1 ± 6.5	-----
10:0-PP (CuSO_4_/H_2_O_2_)	97.78 ± 0.3	<30
10:0.5-PP (CuSO_4_/H_2_O_2_)	95.05 ± 0.7	<30
10:1-PP (CuSO_4_/H_2_O_2_)	80.26 ± 12.9	-----
PQA-DA-PP	99.9	96.17 ± 1.1

## Data Availability

Data will be made available by the corresponding author upon request.

## Supplementary Materials

The following supporting information can be downloaded at: https://www.mdpi.com/article/10.3390/polym16040503/s1, Supplementary Materials (polymers).pdf for Figures S1–S13 and Tables S1–S3. Figure S1: The ^1^H-NMR spectra of TFADA; Figure S2: The ^1^H-NMR spectra of TFADAAC; Figure S3: The ^1^H-NMR spectra of DAAC; Figure S4: The ^1^H-NMR spectra of BrDAAC; Figure S5: The ^1^H-NMR spectra of BrDA; Figure S6: The ^1^H-NMR spectra of DMAEMA-C8; Figure S7: The ^1^H-NMR spectra of PQA-C8; Figure S8: The SEM micrographs of the bare Ti substrate and different modified Ti ones; Figure S9: The SEM micrographs of the bare PP substrate and different modified PP ones; Figure S10: The N1s curve fitting results for the bare Ti substrate and different modified ones; Figure S11: The N1s curve fitting results for the bare PP substrate and different modified ones; Figure S12: The C1s curve fitting results for the bare Ti substrate and different modified ones; Table S1: The C1s curve-fitting results of titanium substrate modified by different methods; Table S2: The C1s curve-fitting results of polypropylene substrate modified by different methods; Table S3: Released copper ion concentration.
