# Supplementary material for: A Facile Surface Modification Scheme for Medical-Grade Titanium and Polypropylene Using a Novel Mussel-Inspired Biomimetic Polymer with Cationic Quaternary Ammonium Functionalities for Antibacterial Application
Source: Polymers (Basel). 2024 Feb 12;16(4):503. doi: 10.3390/polym16040503 (PMC10893476; doi:10.3390/polym16040503)
Supplement: Supplementary file 1 [file polymers-16-00503-s001.zip › polymers-2805718-supplementary.pdf]

A facile surface modification scheme for medical-grade titanium and polypropylene using a novel mussel-inspired biomimetic polymer with cationic quaternary ammonium functionalities for antibacterial application

Chi-Hui Cheng[1], Xiang-Zhen Zeng[2], Wen-Yuan Chiu[2], Jui-Che Lin[2,3,4*]

[1]Department of Pediatrics, College of Medicine, Chang Gung University, Chang Gung Memorial Hospital, Taoyuan, Taiwan. E-mail: pedneph.cheng@msa.hinet.net (CHC)

[2]Department of Chemical Engineering, National Cheng Kung University, Tainan, Taiwan. E-mail: q70w753753@gmail.com (XZZ); chiu1999.vivian@gmail.com (WYC); jclin@mail.ncku.edu.tw (JCL)

[3]Institute of Oral Medicine, [4]School of Dentistry, College of Medicine, National Cheng Kung University, Tainan, Taiwan. E-mail: jclin@mail.ncku.eud.tw (JCL)

Corresponding Author

* Prof. Jui-Che Lin: Department of Chemical Engineering, National Cheng Kung University, Tainan, TAIWAN 70101, Phone: +886-6-275-7575 ext. 62665, Fax: +886-6-234-4996, Email: jclin@mail.ncku.edu.tw ORCID ID: https://orcid.org/0000-0003-4436-655X

S1. Synthesis of TFADA

The ^{1}H-NMR spectra of TFADA were shown in Figure S1 ^{1}H-NMR (500MHz, DMSO-d6) δ (ppm)：9.42 - 9.40 (t, 1H), 8.73 – 8.65 (d, 2H), 6.65 – 6.63 (d, 1H), 6.58 (d, 1H), 6.44 – 6.42 (d, 1H), 3.33 – 3.29 (q, 2H), 2.62 – 2.59 (t, 2H).

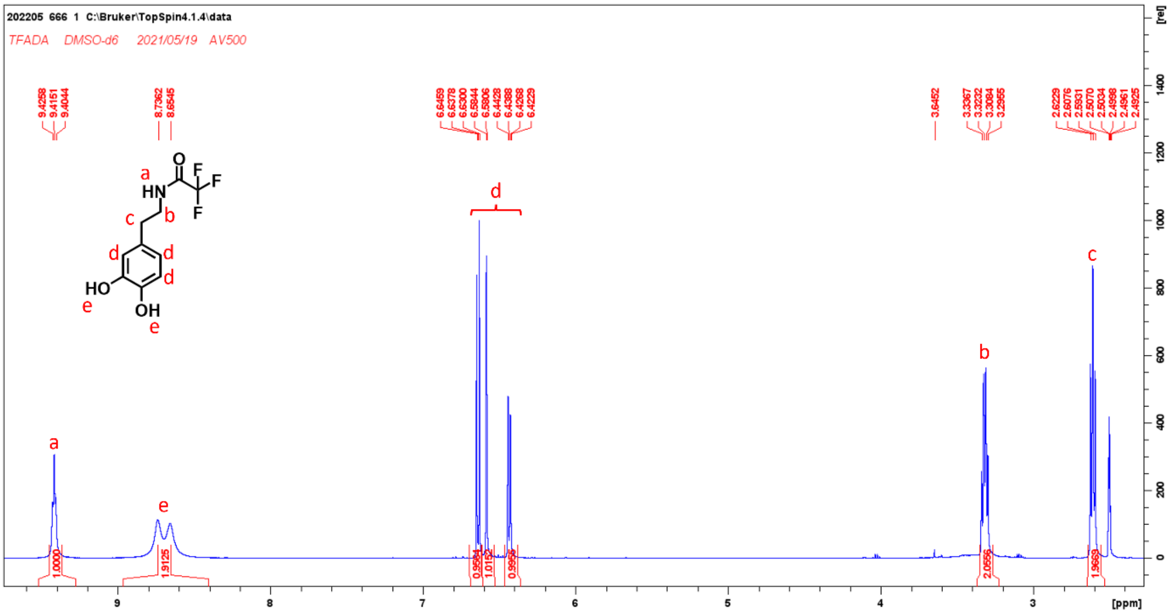

Figure S1 The ^{1}H-NMR spectra of TFADA

S2. Synthesis of TFADAAC

The ^{1}H-NMR spectra of TFADAAC was shown in Figure S2. ^{1}H-NMR (500MHz, $CDCl_3$) δ (ppm)：6.67 – 6.58 (q, 1H), 6.58 – 6.53 (m, 2H), 6.40 – 6.37 (s, 1H), 3.57 – 3.53 (q, 2H), 2.79 – 2.76 (t, 2H), 1.67 – 1.64 (s, 6H).

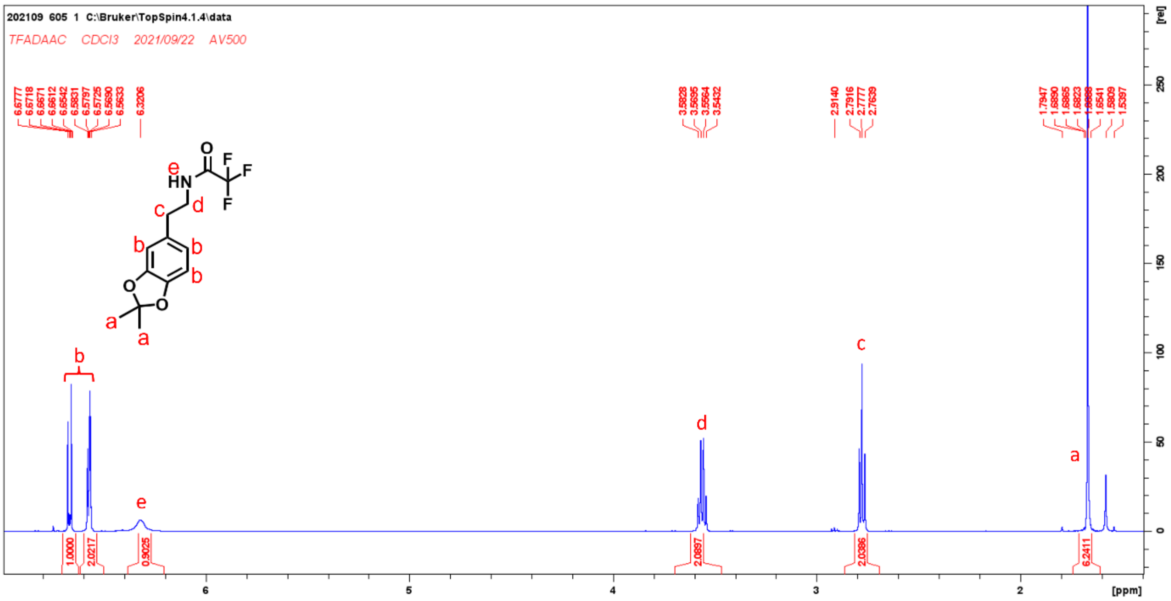

Figure S2 The ^{1}H-NMR spectra of TFADAAC

S3. Synthesis of DAAC

The ^{1}H-NMR spectra of DAAC was shown in Figure S3. ^{1}H-NMR (500MHz, $CDCl_3$) δ (ppm)：6.60 – 6.54 (m, 1H), 6.54 – 6.52 (m, 2H), 3.10 – 2.99 (s, 2H), 2.98 – 2.85 (t, 2H), 2.65 – 2.61 (t, 2H), 1.61 – 1.59 (s, 6H).

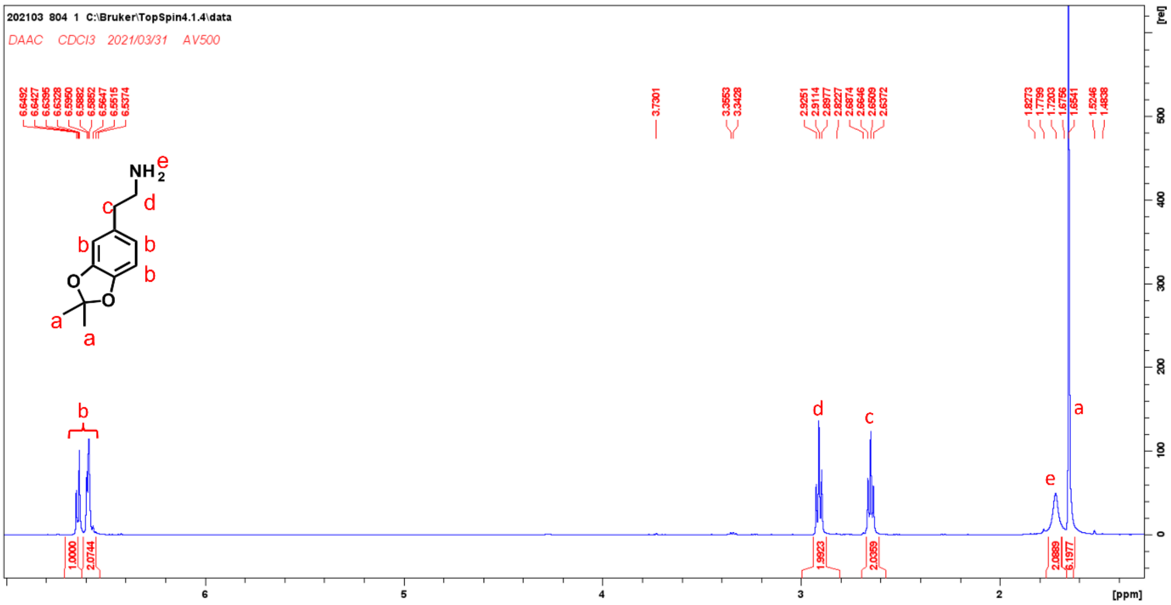

Figure S3 The ^{1}H-NMR spectra of DAAC

S4. Synthesis of BrDAAC

The ^{1}H-NMR spectra of BrDAAC was shown in Figure S4. ^{1}H-NMR (500MHz, $CDCl_3$) δ (ppm)：6.74 – 6.72 (s, 1H), 6.66 – 6.65 (m, 1H), 6.60 – 6.59 (m, 2H), 3.47 – 3.44 (q, 2H), 2.75 – 2.72 (t, 2H), 1.93 – 1.92 (s, 6H), 1.66 – 1.59 (s, 6H).

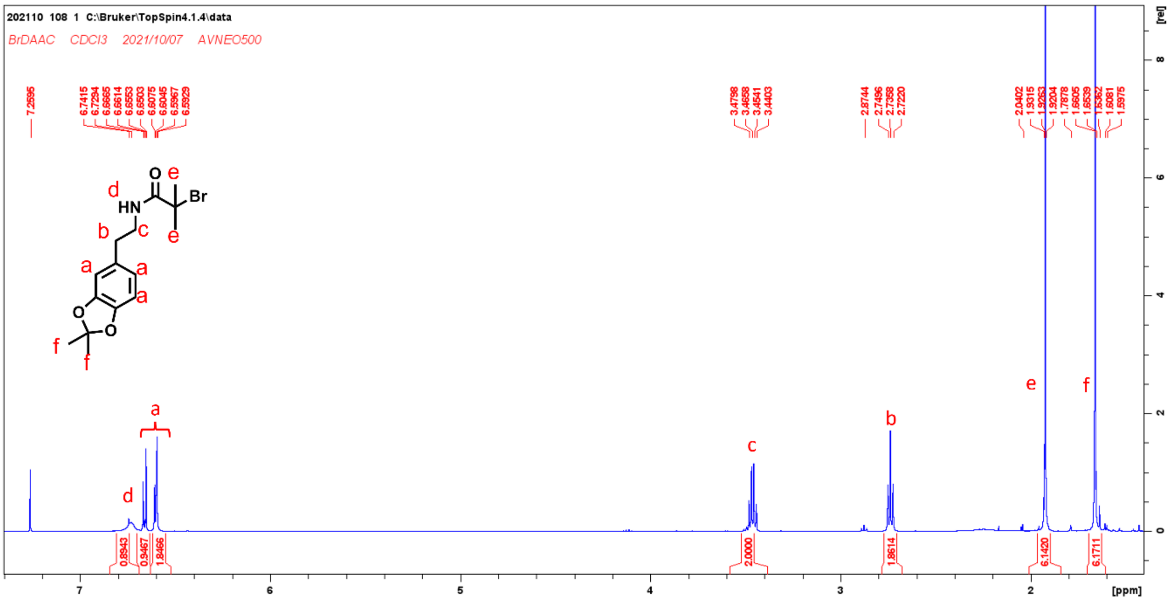

Figure S4 The ^{1}H-NMR spectra of BrDAAC

S5. Synthesis of BrDA

The ^{1}H-NMR spectra of BrDA was shown in Figure S5. ^{1}H-NMR (500MHz, $CDCl_3$) δ (ppm)：6.94 (s, 1H), 6.85 – 6.58 (m, 3H), 5.66 (s, 2H), 3.50 – 3.46 (q, 2H), 2.73 – 2.70 (t, 2H), 1.91 – 1.88 (s, 6H).

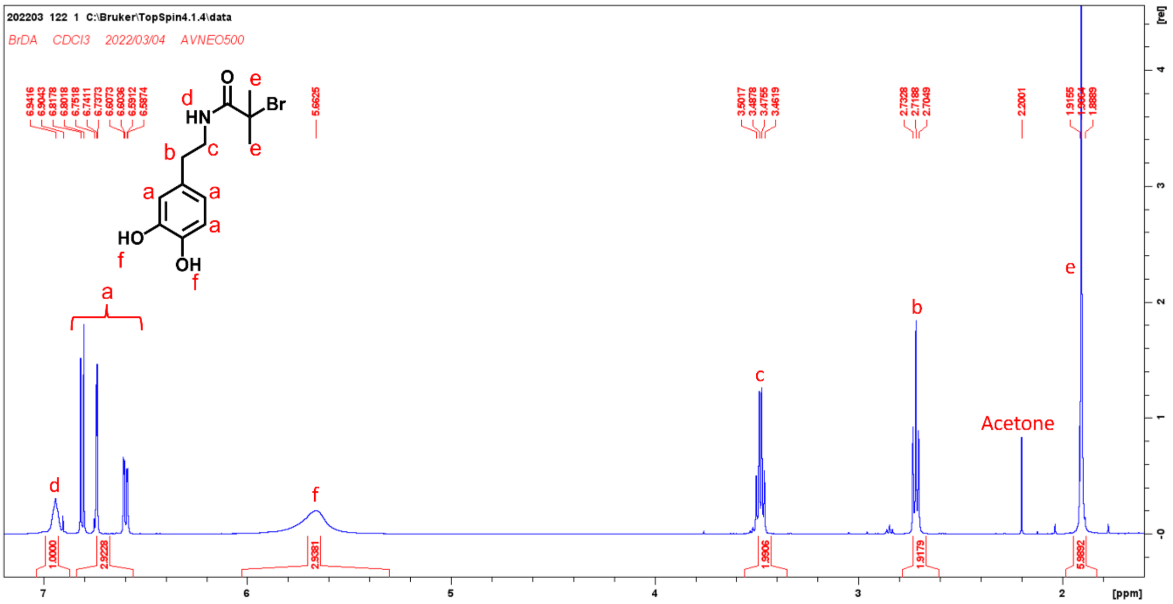

Figure S5 The ^{1}H-NMR spectra of BrDA

S6. Synthesis of DMAEMA-C8

The ^{1}H-NMR spectra of DMAEMA-C8 was shown in Figure S6. ^{1}H-NMR (500MHz, D_2O) δ (ppm)：6.12 (s, 1H), 5.75 – 5.74 (s, 1H), 4.58 (s, 2H), 3.73 – 3.71 (quintet, 2H), 3.36 – 3.33 (quintet, 2H), 3.12 – 3.08 (s, 6H), 1.90 (s, 3H), 1.76 – 1.73 (t, 2H), 1.30 – 1.23 (m, 10H), 0.83 – 0.80 (t, 3H).

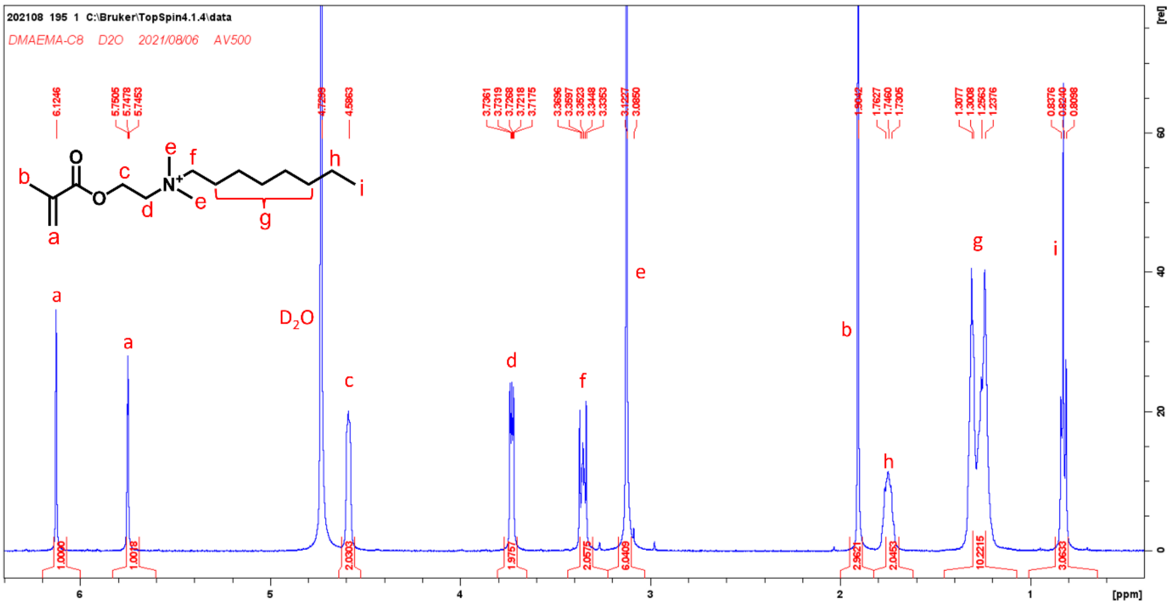

Figure S6 The ^{1}H-NMR spectra of DMAEMA-C8

S7. Synthesis of final product PQA-C8

The ^{1}H-NMR spectra of PQA-C8 was shown in Figure S7. It indicated that the PQA-C8 was prepared successfully. ^{1}H-NMR (500MHz, CD_3OD) δ (ppm)：6.65 – 6.68 (m, 3H), 4.60 (s, 2H), 3.97 (s, 2H), 3.61 (s, 2H), 3.34 (s, 6H), 0.94 – 0.91 (t, 3H).

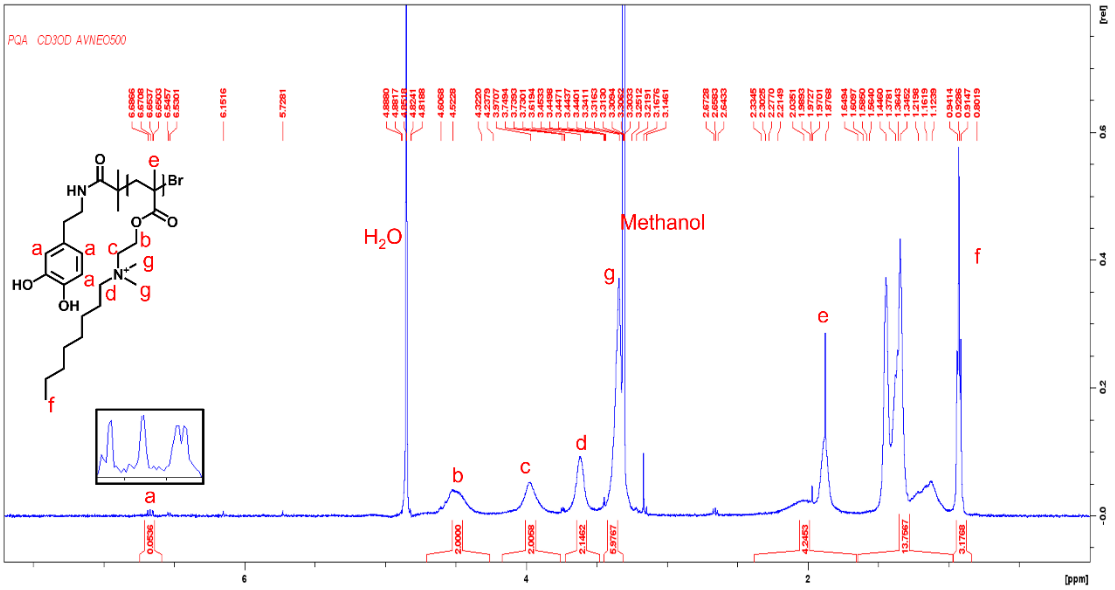

Figure S7 The ^{1}H-NMR spectra of PQA-C8

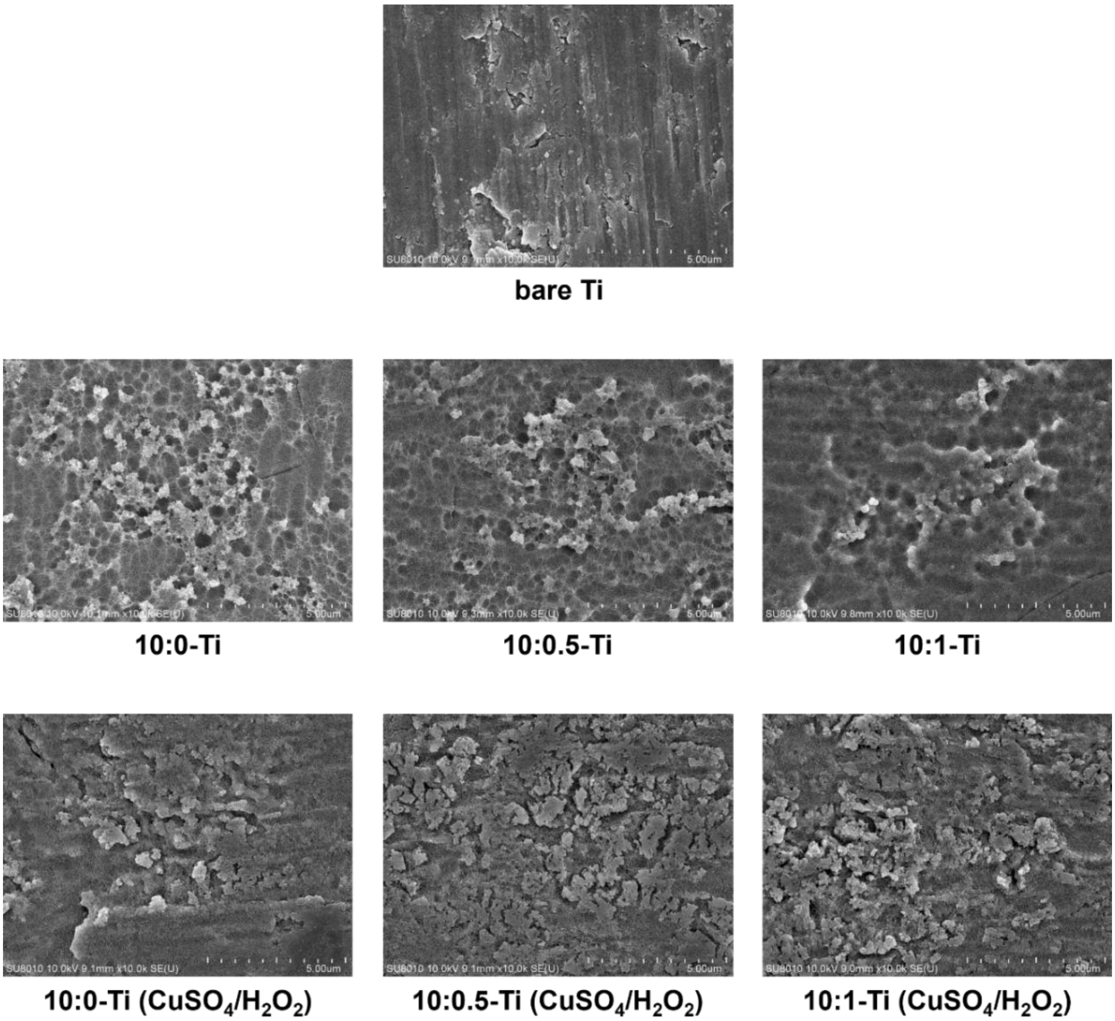

Figure S8 The SEM micrographs of the bare Ti substrate and different modified Ti ones

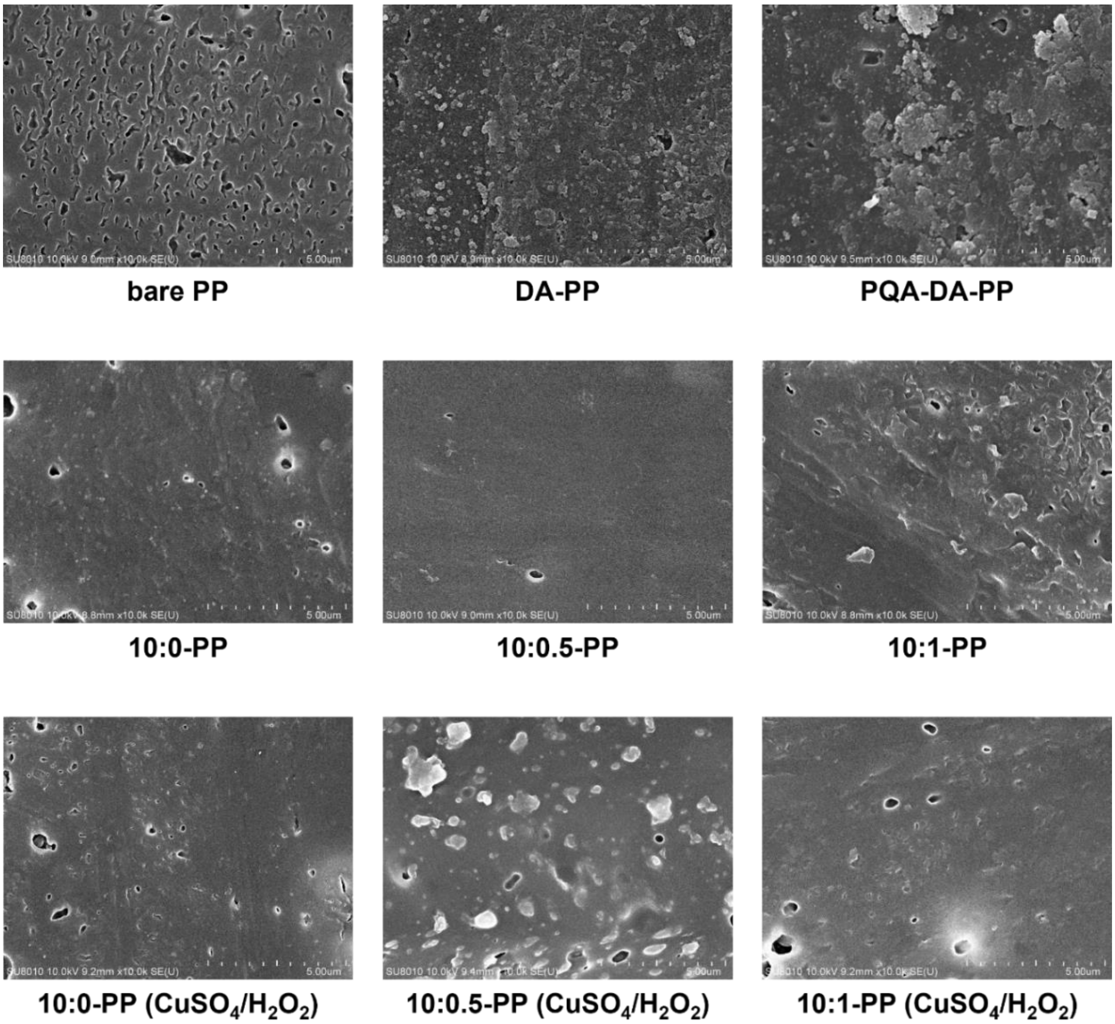

Figure S9 The SEM micrographs of the bare PP substrate and different modified PP ones

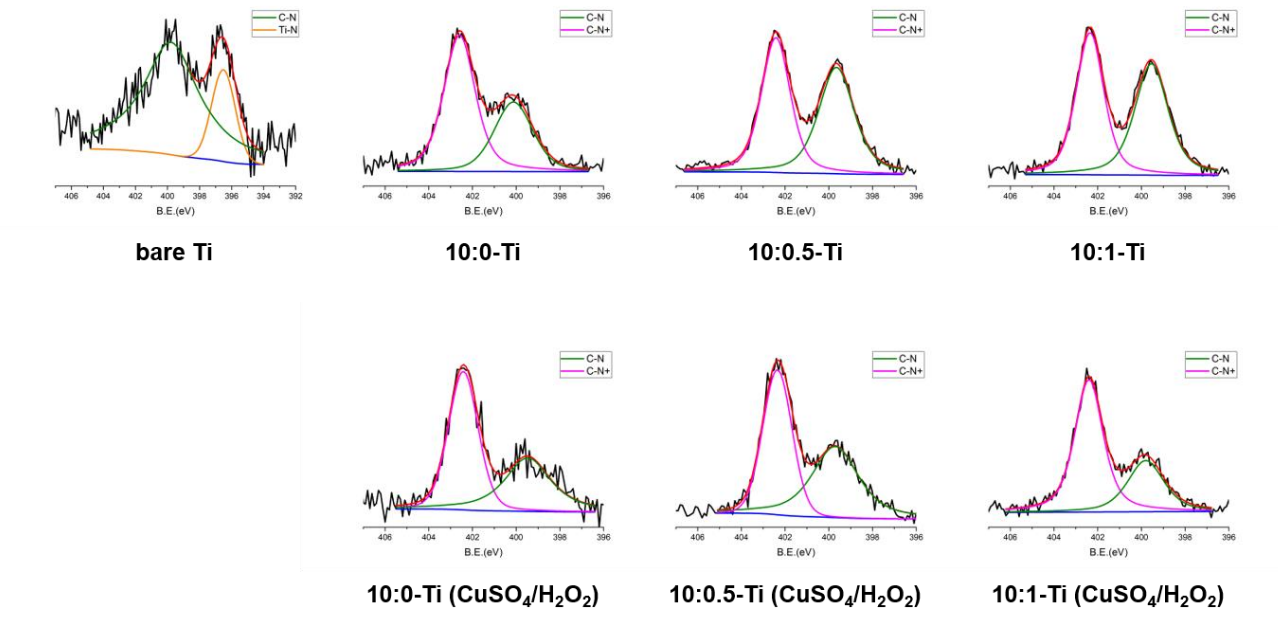

Figure S10 The N1s curve fitting results for the bare Ti substrate and different modified ones

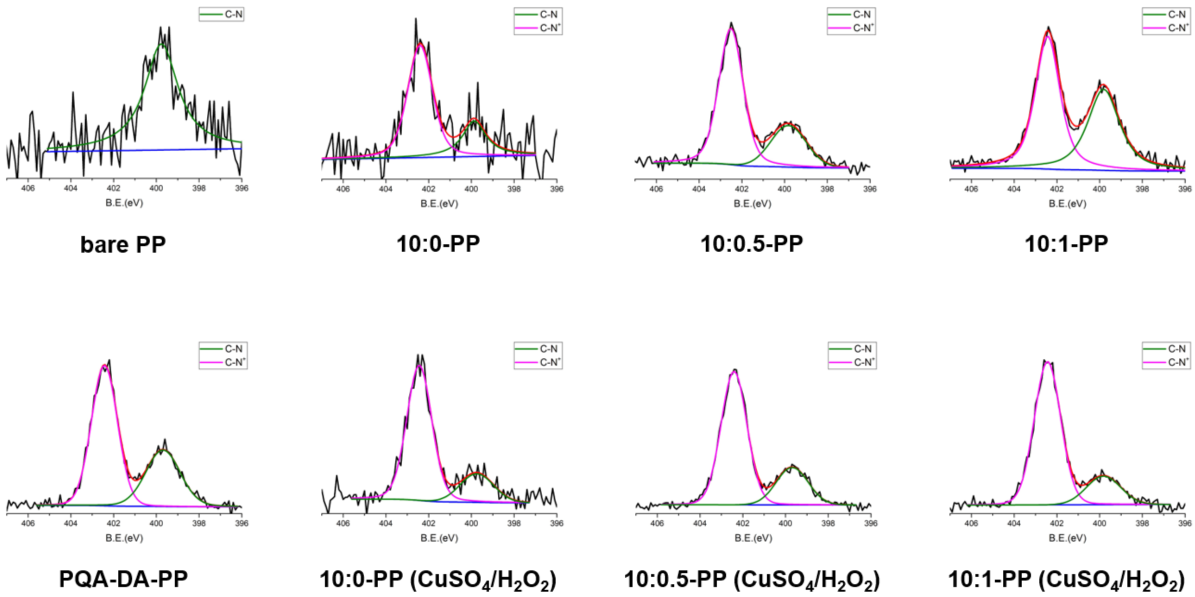

Figure S11 The N1s curve fitting results for the bare PP substrate and different modified ones.

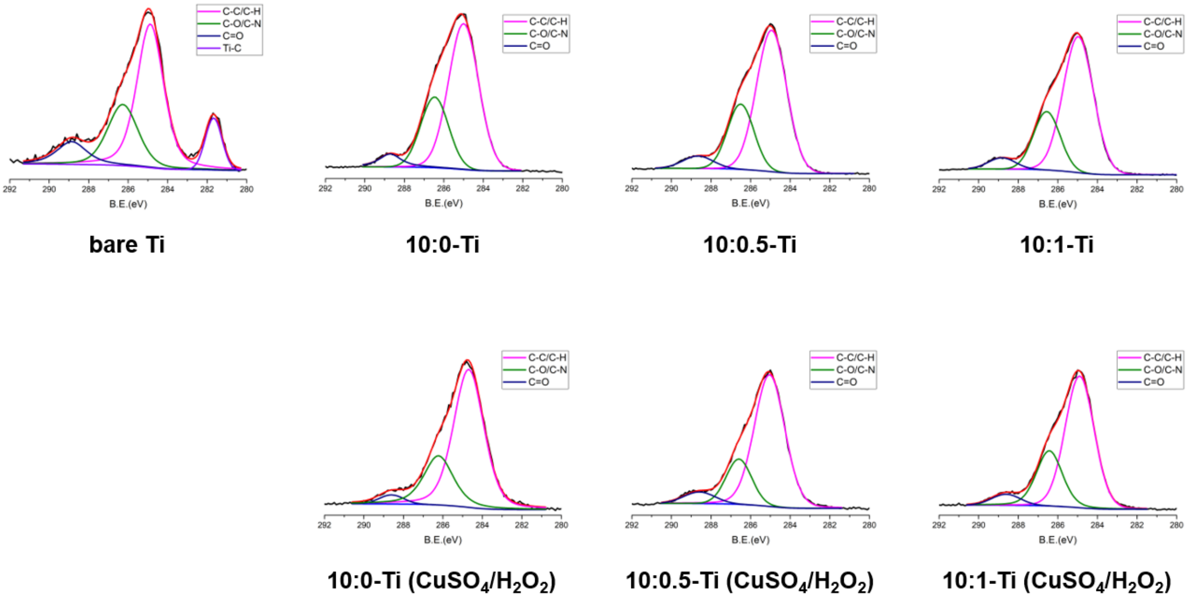

Figure S12 The C1s curve fitting results for the bare Ti substrate and different modified ones.

Table S1 The C1s curve-fitting results of titanium substrate modified by different methods

**Sample**	**C-C/C-H (285 eV)**	**C-O/C-N (286.6 eV)**	**C=O (288.6 eV)**	**Ti-C (282 eV)**
bare Ti	53.9%	25.2%	10.6%	10.3%
10:0-Ti	62.6%	31.8%	5.7%	0%
10:0.5-Ti	67.3%	26.8%	5.9%	0%
10:1-Ti	69.3%	25.7%	5.0%	0%
10:0-Ti ($CuSO_4/H_2O_2$)	70.1%	26.6%	3.3%	0%
10:0.5-Ti ($CuSO_4/H_2O_2$)	73.3%	20.5%	6.3%	0%
10:1-Ti ($CuSO_4/H_2O_2$)	67.5%	27.1%	5.4%	0%

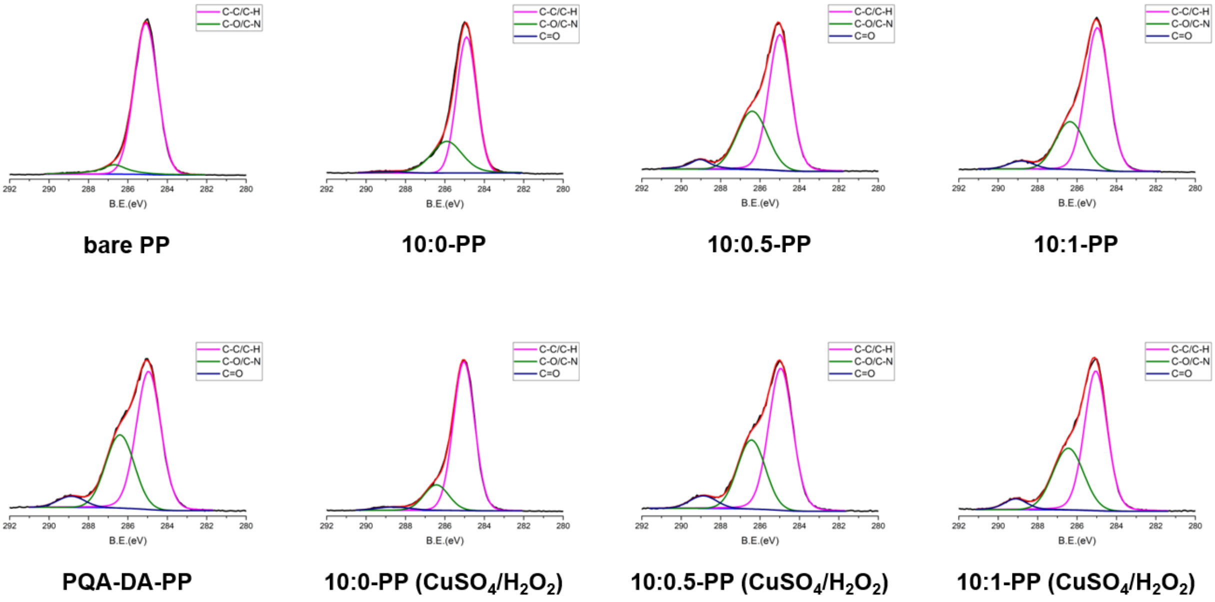

Figure S13 The C1s curve fitting results for the bare PP substrate and different modified ones.

Table S2 The C1s curve-fitting results of polypropylene substrate modified by different methods

**Sample**	**C-C/C-H (285 eV)**	**C-O/C-N (286.6 eV)**	**C=O (288.6 eV)**
bare PP	91.2 %	8.8 %	0.0%
10:0-PP	70.2 %	28.6 %	1.2%
10:0.5-PP	60.8 %	34.0 %	5.2%
10:1-PP	68.7 %	27.3 %	4.0%
10:0-PP ($CuSO_4/H_2O_2$)	81.1 %	16.1 %	2.8%
10:0.5-PP ($CuSO_4/H_2O_2$)	61.4 %	32.9 %	5.7%
10:1-PP ($CuSO_4/H_2O_2$)	60.8 %	34.1 %	5.1%
PQA-DA-PP	59.85 %	35.13 %	5.02%

Table S3 Released copper ion concentration

Sample	Cu^{2+} concentration (ppm)	RSD%
10:0-Ti ($CuSO_4/H_2O_2$)	0.055	1.93
10:0.5-Ti ($CuSO_4/H_2O_2$)	0.039	1.13
10:1-Ti ($CuSO_4/H_2O_2$)	0.037	2.73
10:0-PP ($CuSO_4/H_2O_2$)	0.029	4.71
10:0.5-PP ($CuSO_4/H_2O_2$)	0.029	1.13
